# Preoperative aspartate aminotransferase-to-platelet ratio index (APRI) is a predictor on postoperative outcomes of hepatocellular carcinoma

**DOI:** 10.1097/MD.0000000000005486

**Published:** 2016-12-02

**Authors:** JiWen Cheng, Pu Zhao, JiangBo Liu, Xi Liu, XuanLin Wu

**Affiliations:** a Department of Pediatric Surgery, The Second Affiliated Hospital of Xi’an Jiaotong University; b Department of Neonatology, Shaanxi Provincial People's Hospital, Xi’an, Shaanxi Province; c Department of General Surgery, First Affiliated Hospital, College of Clinical Medicine, Henan University of Science and Technology, Luoyang, Henan Province; d Department of Pathology, First Affiliated Hospital of Xi’an Jiaotong University, Xi’an, Shaanxi Province, China.

**Keywords:** APRI, biochemical marker, hepatectomy, hepatocellular carcinoma, short-term outcomes

## Abstract

Preoperative aspartate aminotransferase-to-platelet ratio index (APRI) has been identified as a biochemical marker for histological fibrogenesis and fibrosis in cirrhosis and prognosis of hepatocellular carcinoma (HCC). Whether preoperative APRI can predict postoperative short-term outcomes has not been studied. The purpose of this study was to investigate the ability of preoperative APRI to predict short-term outcomes following liver resection for HCC. APRI was evaluated in 360 patients undergoing liver resection for HCC. The receiver operating characteristic curve analysis was conducted to determine the cutoff value of the APRI in predicting postoperative morbidity. Univariate and multivariate analysis was performed to identify the risk factors for postoperative outcomes. The correlation of the preoperative APRI value with clinicopathological parameters was also examined. We found that the optimal cutoff value of the APRI was set at 9.5 for postoperative complications. APRI was an independent risk factor for overall complications by univariate and multivariate analyses. HCC patients with elevated APRI (>9.5) had a worse liver function and significantly higher postoperative complication rate. In conclusion, preoperative APRI is a useful biochemical marker to predict postoperative outcomes in HCC patients.

## 1 Introduction

Hepatocellular carcinoma (HCC) is the most common primary malignancy of the liver and the third most frequent cause of cancer-related deaths worldwide.^[1]^ The management of liver cancer has improved significantly in the last few decades. Nevertheless, surgery still remains the first choice of curative treatment for HCC so far.^[2]^ Notably, the posthepatectomy complication rate remains high, especially for HCC patients with underlying advanced liver fibrosis and cirrhosis.^[3]^ It has been reported that postoperative complications are predictive of poor prognosis in HCC.^[4]^ Therefore, it is extremely important to identify patient subpopulations at a high risk of adverse postoperative outcomes to optimize postoperative rational treatments and provide treatments to them without delay.

Recently, preoperative aspartate aminotransferase (AST)-to-platelet count ratio index (APRI) has been identified as a biochemical marker for histological fibrogenesis and fibrosis in cirrhosis^[5,6]^ and poor prognosis of HCC.^[7]^ Postoperative complications are positively related to the prognosis of HCC patients. However, it remains unclear whether preoperative APRI can also serve as a biomarker that can predict short-term HCC in patients who undergo curative hepatectomy. In the present study, we investigated the role of preoperative APRI to predict the postoperative short-term outcomes in patients with HCC.

## 2 Methods

### 2.1 Patients and determination of APRI

From the beginning of 2009 to the end of 2014, liver resection for HCC was performed for 360 patients who met the Milan criteria in First and Second Affiliated Hospital of Medical College, Xi’an Jiaotong University, were enrolled onto this study. All patients had complete clinical and laboratory data. To ensure that platelet count was representative of normal baseline values, HCC patients with coexistent hematologic disorders were excluded. Informed consents were obtained from all of the patients, and this study was conducted in accordance with the Helsinki Declaration and the guidelines of the Ethics Committee at Xi’an Jiaotong University. The APRI was calculated based on laboratory data at the time of HCC diagnosis as ([AST/ULN∗]/platelet count) × 100.^[8]^

### 2.2 Definition of outcomes

The surgical complications were classified according to the modified Clavien classification.^[9,10]^ Major complication was defined as grade 3 or above. The median of blood loss and massive hemorrhage was defined as the blood loss of 500 mL and more than 500 mL, respectively.

### 2.3 Statistical analysis

Statistical analysis was performed by SPSS 20.0 software (SPSS, Chicago, IL). Comparisons between groups were analyzed using Student *t* test or the Mann–Whitney *U* test. The categorical data were compared with the χ^2^ test or Fisher exact test as appropriate. A receiver operating characteristic (ROC) curve was constructed to determine the optimal cutoff of APRI in predicting postoperative morbidity. The optimal cutoff value was set as the value maximizing the sum of sensitivity and specificity. Variables associated with the development of postoperative complication were first assessed using a univariate analysis, and then the variables with *P* value less than 0.05 were subjected to multivariate logistic regression analysis to identify the independent predictors for the development of postoperative complication. *P* < 0.05 was considered significant.

## 3 Results

### 3.1 Patient characteristics and APRI

A total of 360 HCC patients were enrolled in this study. This population comprised 297 males and 63 females with a mean age of 53.36 ± 13.84 years. All the patients underwent open hepatectomy, 338 (93.89%) and 22 (6.11%) had background B viral hepatitis (HBV) and C viral hepatitis (HCV), respectively. Of the patients, 138 (38.33%) received liver resection of 1 segment, 148 (41.11%) received liver resection of 2 segments, and 74 (20.56%) received liver resection of 3 segments. The median APRI value was 9.50 (1.00–151.25) (mean ± SD, 13.75 ± 16.75). Details of these features are shown in Table 1.

**Table 1:**
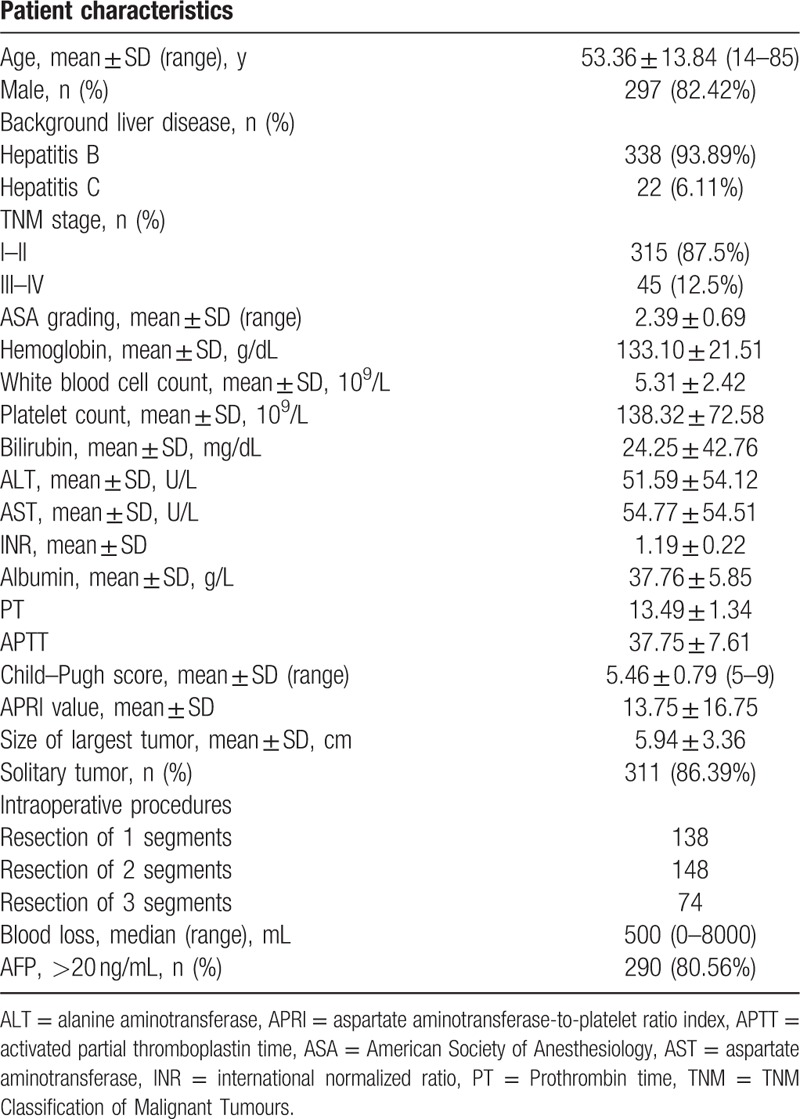
Baseline characteristics.

### 3.2 Prediction of postoperative complications

**Figure 1:**
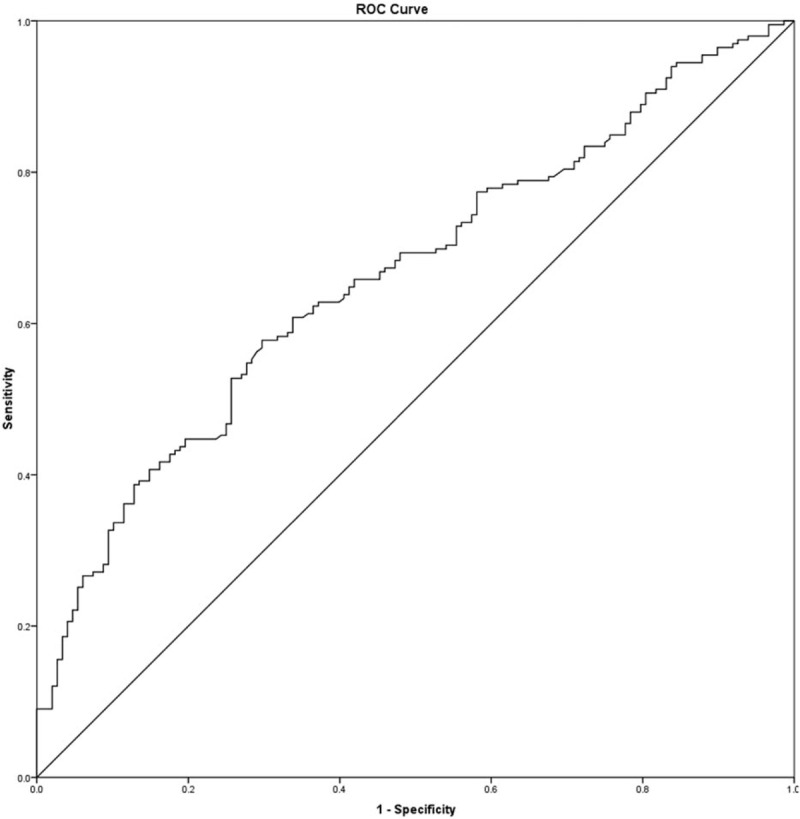
Receiver operating characteristic curves for aspartate aminotransferase-to-platelet count ratio index (APRI) in relation to postoperative complication. AUROC curves were 0.663 (95% confidence interval, 0.61–0.72; *P* < 0.001) for APRI. The calculated cutoff value for APRI was 9.5, with sensitivity of 57.8%, specificity of 70.3% in the prediction of postoperative complications.

**Figure 2:**
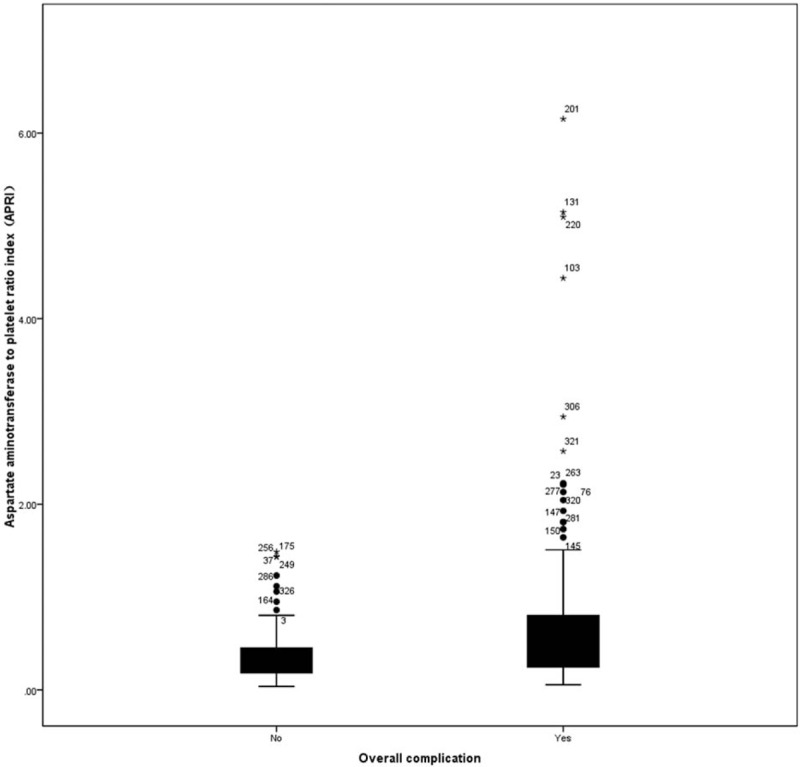
Distribution of aspartate aminotransferase-to-platelet count ratio index according to the occurrence of postoperative complication.

Table 2 shows the classification of complications. Among the 360 patients enrolled in this study, postoperative complications occurred in 206 patients (57.22%) and major complications occurred in 27 patients (7.5%). The ROC curves for APRI in relation to postoperative complication are shown in Fig. 1. The area under the receiver operating characteristic (AUROC) curves were 0.66 (95% confidence interval [CI], 0.61–0.72; *P* < 0.001). The calculated cutoff value for APRI was 9.5, with a sensitivity of 57.8% and a specificity of 70.3% in the prediction of complications. The distribution of APRI values according to the occurrence of postoperative complication is shown in Fig. 2. In addition, Spearman correlation analysis showed that the APRI values were inversely correlated with intraoperative blood loss (*r* = −0.15, *P* = 0.004).

**Table 2:**
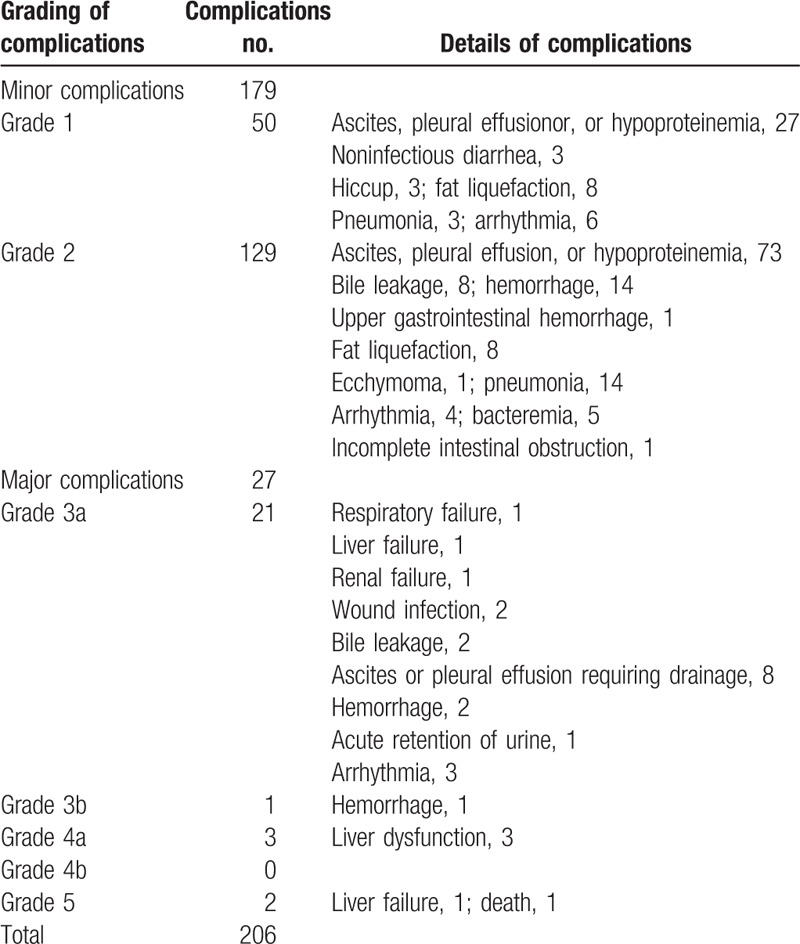
Postoperative complications according to the modified Clavien classification.

### 3.3 Risk factors of postoperative complications

The results of univariate and multivariate analyses of the predictors of postoperative overall complications are shown in Table 3. On admission, AST (*P* < 0.001), alanine aminotransferase (ALT) (*P* = 0.002), bilirubin (*P* = 0.002), albumin (*P* < 0.001), APRI (*P* < 0.001), prothrombin time (PT) (*P* < 0.001), international normalized ratio (INR) (*P* < 0.001), blood loss (*P* < 0.001), blood transfusion (*P* = 0.001), American Society of Anesthesiology (ASA) grading (*P* = 0.001), and operation time (*P* < 0.001) were significant associated with postoperative complications. Multivariate analysis showed that the 4 independent predictors of postoperative complications in the final model were age (*P* = 0.032; odds ratio 1.022; 95% CI, 1.001–1.043), INR (*P* = 0.001; odds ratio 63.180; 95% CI, 5.230–761.840), APRI (*P* < 0.001; odds ratio 4.229; 95% CI, 1.935–1.327), and blood loss (*P* < 0.001; odds ratio 1.001; 95% CI, 1.000–1.001) remained as independent predictors.

**Table 3:**
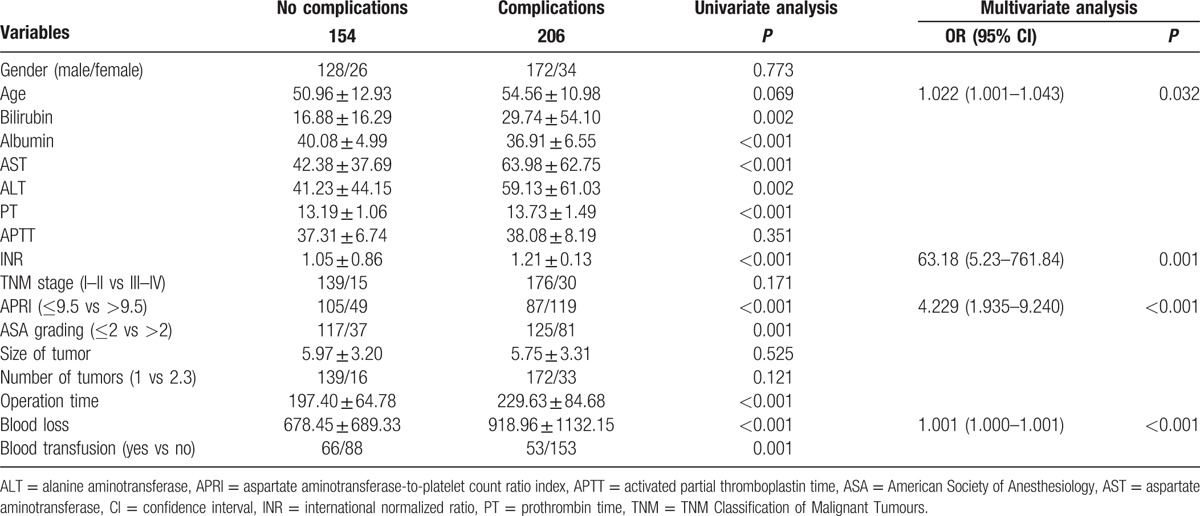
Univariate and multivariate logistic regression analysis of postoperative complications.

### 3.4 APRI and clinicopathological characteristics of HCC patients

We compared the baseline characteristics of the 192 patients with a low APRI (<9.5) with the 168 patients whose APRI was high (>9.5) (Table 4). The results revealed that patients with elevated APRI = 9.5 (>9.5) had a higher Child–Pugh score; higher preoperative levels of ALT, AST, bilirubin, albumin, INR, and more volume of blood loss; longer postoperative hospital stay; longer portal vein interrupt; frequent occurrence of complications; and lower albumin. Furthermore, the Spearman correlation analysis showed that APRI was significantly associated with the ALT (*r* = 0.602, *P* < 0.001), AST (*r* = 0.772, *P* < 0.001), bilirubin (*r* = 0.307, *P* < 0.001), albumin (*r* = −0.256, *P* < 0.001), PT (*r* = 0.131, *P* = 0.014), INR (*r* = 0.212, *P* < 0.001), blood loss (*r* = 0.218, *P* < 0.001), Child–Pugh score (*r* = 0.218, *P* < 0.001), ASA grading (*r* = 0.159, *P* = 0.003), and portal vein interrupt (*r* = 0.114, *P* = 0.033) (Table 5).

**Table 4:**
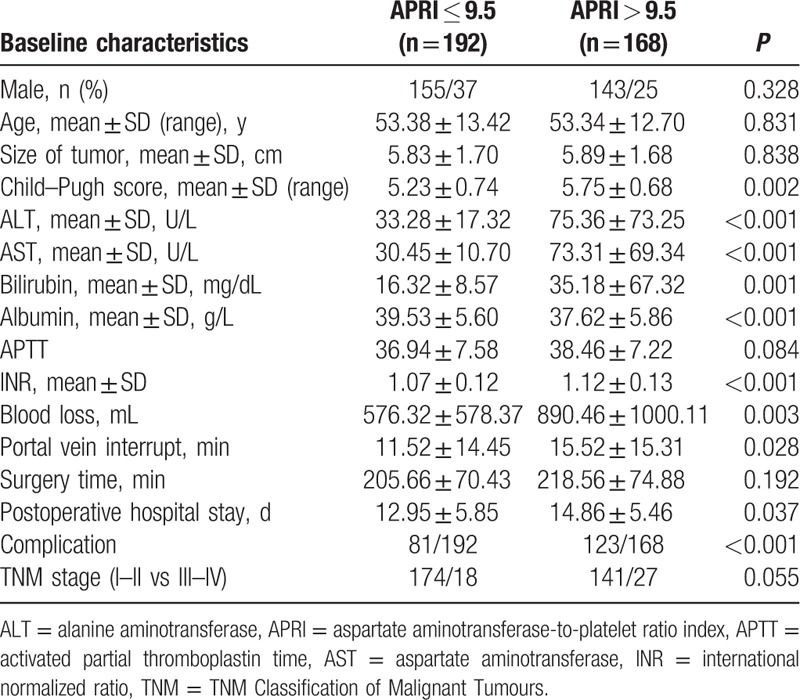
Baseline comparison between patients with APRI ≤ 9.5 and >9.5.

**Table 5:**
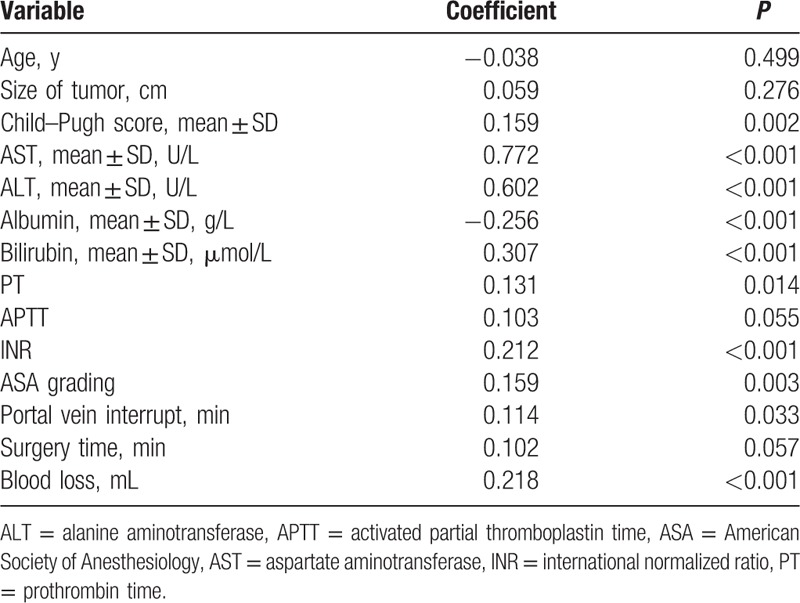
Spearman correlation analysis between APRI and clinical characteristics.

### 3.5 Discussion

Despite significant advancement in surgical technique and perioperative care in recent decades, the mortality rate after hepatectomy remains high, especially in HCC patients with chronic liver disease.^[11–13]^ Some preoperative assessments had been reported, such as preoperative portal pressure, technetium 99m-labeled asialoglycoprotein analog, indocyanine green (ICG) retention test, and computed tomography (CT) liver volumetry are useful for predicting prognosis before hepatectomy.^[14–16]^ Nevertheless, these assessments were less effective for detecting early hepatic impairment, especially for patients with advanced liver fibrosis or early liver cirrhosis.^[17,18]^ It is worth noting that degree of liver fibrosis was confirmed as a negative predictor of liver regeneration and restoration of liver function after liver resection.^[19]^ The previous studies^[17–20]^ also reported that the liver histological and fibrosis are 2 main risk of postoperative complications; however, these biomarkers are always difficult to be examined and waste of time. In addition, advanced hepatic fibrosis or cirrhosis has a close relationship with the mortality and development of postoperative complications, such as ascites, liver failure, and worsening encephalopathy.^[17,20]^ It has been established that liver stiffness measurement (LSM) using transient elastography is a novel method for detection of liver fibrosis and cirrhosis with high accuracy. Wong et al^[9]^ reported high LSM (LSM > 12.0 kPa) predicted worse posthepatectomy outcomes, and preoperative LSM was better than ICG test in the prediction of major postoperative complications. Furthermore, hepatic stiffness measurement has been widely used *in clinical* trials because of it could *avoid* liver *biopsy in most patients.* Nevertheless, hepatic stiffness measurement is not routinely assessed for HCC before liver resection all over the world for it is expensive.^[21]^

It has been reported that APRI is correlated with histologic degree of liver fibrosis and cirrhosis.^[8,22]^ Ichikawa et al^[23]^ reported that preoperative APRI independently predicted hepatic failure following liver resection for HCC, and patients with an APRI of 10 or more have a high risk of postoperative hepatic failure. In the present study, we identified APRI was an independent risk factor for overall postoperative complications by univariate and multivariate analyses. We found that HCC patients with elevated APRI (>9.5) had a worse liver function and significantly higher postoperative complication rate. Obviously, there was difference between the optimal cutoff value of the APRI for postoperative complications and postoperative hepatic failure in the present study and the previous report.^[24]^ The different geographical areas and different HCC etiologies may contribute to the difference. Notably, HCC patients with background of HBV and HCV were enrolled in the present study and the previous report, respectively. Although the previous study has investigated the function of APRI predicting hepatic failure following liver resection for HCC, it has not observed the detailed parameters, such as albumin, international normalized ratio, and portal vein interrupt, which were proved to be associated with the changes of APRI levels. Moreover, the results of the present study first revealed that APRI is a useful index to predict postoperative outcomes in HCC patients with background of HBV.

APRI is commonly used to determine the extent of hepatic fibrosis in chronic hepatitis C virus,^[8]^ while Ray Kim et al^[24]^ reported that APRI scores are not suitable for use in clinical practice in chronic hepatitis B patients for assessment of hepatic fibrosis recently. Thus, further studies are necessary to validate the value of APRI for predicting postoperative complications in HBV. Our results also showed that HCC patients with elevated APRI had a more volume of blood loss. The volume of intraoperative blood loss has been shown to have significant negative impacts on postoperative mortality and long-term survival outcomes.^[25,26]^ Shen et al^[7]^ reported preoperative APRI was associated with adverse characteristic features and poor prognosis in HCC. In addition, the content of resection is another very significant factor related with the postoperative complications. Only patients with HCC within the Milan criteria were enrolled in the present study, because previous study has addressed the predictive value of the content of resection regarding liver function and complications after major liver resection.^[9]^

Taken together, because liver function was a well known risk factor for postoperative complications, the result of higher postoperative complication rate and poor prognosis in HCC patient with higher APRI could be explained by the presence of worse liver function to a certain extent. Thus, preoperative determination of the APRI could inform the surgeon about residual liver function and identify patients at a high risk of adverse postoperative outcomes to optimize postoperative rational treatments. However, further clinicoimmunologic studies are needed to confirm the significance of preoperative APRI to predict postoperative complications in HCC patients with different HBV and/or HCV infection status.

## 4 Conclusion

This retrospective analysis showed that preoperative APRI was correlated with postoperative complications and may be used clinically to identify HCC patients at increased risk for adverse postoperative outcomes.

## Acknowledgments

We would like to thank the residents and nursing staff of the Department of Hepatobiliary Surgery and General Surgery for their contributions.
